# Supplementary material for: An open-source framework for large-scale, flexible evaluation of biomedical text mining systems
Source: J Biomed Discov Collab. 2008 Jan 29;3:1. doi: 10.1186/1747-5333-3-1 (PMC2276192; doi:10.1186/1747-5333-3-1)
Supplement: Additional file 1 — Evaluation platform source code. The additional file contains the software evaluation framework discussed in this paper. The most current release can be downloaded from SourceForge.net [1]. See the accompanying README file for instructions on installation and use. Also included in the distribution is a collection of UIMA wrappers for some commonly used BioNLP tools and annotated corpora. [file 1747-5333-3-1-S1.zip › bionlp_uima_repository-1.0.1/docs/api/src-html/edu/uchsc/ccp/util/nlp/stats/PRFResult.html]

```
001    package edu.uchsc.ccp.util.nlp.stats;
002    
003    import java.io.PrintStream;
004    import java.util.ArrayList;
005    import java.util.List;
006    
007    import edu.uchsc.ccp.util.nlp.annotation.TextAnnotation;
008    
009    /**
010     * This class is a data structure for cataloging the results of an NLP experiment. Not only are TP, FP, and FN counts logged, but the annotations that
011     * were responsible for each TP, FP, and FN count are also recorded.
012     * 
013     * @author William A Baumgartner, Jr.
014     * 
015     */
016    public class PRFResult {
017    
018        private double precision;
019    
020        private double recall;
021    
022        private double fmeasure;
023    
024        private int truePositiveCount;
025    
026        private int falsePositiveCount;
027    
028        private int falseNegativeCount;
029    
030        /* These lists are used to store the annotations responsible for the TP, FP, and FN counts. */
031        private List<TextAnnotation> tpAnnotations;
032    
033        private List<TextAnnotation> fpAnnotations;
034    
035        private List<TextAnnotation> fnAnnotations;
036    
037        /* A textual description of the NLP experiment cataloged by this PRFResult object */
038        private String title;
039    
040        /**
041         * Initialize a new PRFResult by inputting counts for true positives, false positives, and false negatives.
042         * 
043         * @param tp
044         *            a count of true positives
045         * @param fp
046         *            a count of false positives
047         * @param fn
048         *            a count of false negatives
049         */
050        public PRFResult(int tp, int fp, int fn) {
051            this.truePositiveCount = tp;
052            this.falsePositiveCount = fp;
053            this.falseNegativeCount = fn;
054    
055            // documentID2AnnotationsMap = new HashMap<String, Map<String, List<TextAnnotation>>>();
056            tpAnnotations = new ArrayList<TextAnnotation>();
057            fpAnnotations = new ArrayList<TextAnnotation>();
058            fnAnnotations = new ArrayList<TextAnnotation>();
059    
060            computePRFStats();
061        }
062    
063        /**
064         * Initialize a new PRFResult by inputting counts for true positives, false positives, and false negatives.
065         * 
066         * @param tp
067         *            a count of true positives
068         * @param fp
069         *            a count of false positives
070         * @param fn
071         *            a count of false negatives
072         * @param title
073         *            a textual description of the NLP experiment under study
074         */
075        public PRFResult(int tp, int fp, int fn, String title) {
076            this.truePositiveCount = tp;
077            this.falsePositiveCount = fp;
078            this.falseNegativeCount = fn;
079            this.title = title;
080    
081            // documentID2AnnotationsMap = new HashMap<String, Map<String, List<TextAnnotation>>>();
082            tpAnnotations = new ArrayList<TextAnnotation>();
083            fpAnnotations = new ArrayList<TextAnnotation>();
084            fnAnnotations = new ArrayList<TextAnnotation>();
085    
086            computePRFStats();
087        }
088    
089        /**
090         * Update the precision, recall, and F-measure statistics for this PRFResult
091         */
092        private void computePRFStats() {
093            this.precision = NLPStats.computePrecision(truePositiveCount, falsePositiveCount);
094            this.recall = NLPStats.computeRecall(truePositiveCount, falseNegativeCount);
095            this.fmeasure = NLPStats.computeFMeasure(truePositiveCount, falsePositiveCount, falseNegativeCount);
096        }
097    
098        public double getFmeasure() {
099            return fmeasure;
100        }
101    
102        public double getPrecision() {
103            return precision;
104        }
105    
106        public double getRecall() {
107            return recall;
108        }
109    
110        public int getFalseNegativeCount() {
111            return falseNegativeCount;
112        }
113    
114        public void setFalseNegativeCount(int falseNegativeCount) {
115            this.falseNegativeCount = falseNegativeCount;
116            computePRFStats();
117        }
118    
119        public int getFalsePositiveCount() {
120            return falsePositiveCount;
121        }
122    
123        public void setFalsePositiveCount(int falsePositiveCount) {
124            this.falsePositiveCount = falsePositiveCount;
125            computePRFStats();
126        }
127    
128        public int getTruePositiveCount() {
129            return truePositiveCount;
130        }
131    
132        public void setTruePositiveCount(int truePositiveCount) {
133            this.truePositiveCount = truePositiveCount;
134            computePRFStats();
135        }
136    
137        /**
138         * Output to the given <code>PrintStream</code> the statistics recorded by this PRFResult
139         * 
140         * @param ps
141         *            the <code>PrintStream</code> to use
142         */
143        public void printStats(PrintStream ps) {
144            ps.println("---------------- " + title + " ----------------");
145            ps.println("#TP:" + truePositiveCount + " #FP:" + falsePositiveCount + " #FN:" + falseNegativeCount);
146            ps.println("----  P: " + this.getPrecision());
147            ps.println("----  R: " + this.getRecall());
148            ps.println("----  F: " + this.getFmeasure());
149            ps.println("----------------------------------------------");
150        }
151    
152        /**
153         * Output to the given <code>PrintStream</code> the annotations responsible for the TP, FP, and FN statistics.
154         * 
155         * @param ps
156         *            the <code>PrintStream</code> to use
157         */
158        public void printAnnotations(PrintStream ps) {
159            for (TextAnnotation ta : tpAnnotations) {
160                ps.println("TP -- " + ta.getSingleLineRepresentation(false, true));
161            }
162            for (TextAnnotation ta : fpAnnotations) {
163                ps.println("FP -- " + ta.getSingleLineRepresentation(false, true));
164            }
165            for (TextAnnotation ta : fnAnnotations) {
166                ps.println("FN -- " + ta.getSingleLineRepresentation(false, true));
167            }
168        }
169    
170        /**
171         * Update this <code>PRFResult</code> object by adding the TP, FP, and FN counts from another <code>PRFResult</code> object. Note, at this
172         * time, the annotations do not get passed along, only the counts. This is for space considerations when evaluating large document collections.
173         * 
174         * @param prf
175         */
176        public void add(PRFResult prf) {
177            this.truePositiveCount = this.truePositiveCount + prf.getTruePositiveCount();
178            this.falsePositiveCount = this.falsePositiveCount + prf.getFalsePositiveCount();
179            this.falseNegativeCount = this.falseNegativeCount + prf.getFalseNegativeCount();
180            computePRFStats();
181    
182        }
183    
184        public String getTitle() {
185            return title;
186        }
187    
188        public void setTitle(String title) {
189            this.title = title;
190        }
191    
192        /**
193         * Retrieve the annotations responsible for the false negative counts
194         * 
195         * @return a list of <code>TextAnnotation</code> objects associated with the false negative counts
196         */
197        public List<TextAnnotation> getFNAnnotations() {
198            return fnAnnotations;
199        }
200    
201        /**
202         * Add a false negative <code>TextAnnotation</code>
203         * 
204         * @param fnAnnotation
205         */
206        public void addFNAnnotation(TextAnnotation fnAnnotation) {
207            fnAnnotations.add(fnAnnotation);
208        }
209    
210        /**
211         * Set the false negative <code>TextAnnotation</code> list
212         * 
213         * @param fnAnnotations
214         */
215        public void setFNAnnotations(List<TextAnnotation> fnAnnotations) {
216            this.fnAnnotations = fnAnnotations;
217        }
218    
219        /**
220         * Retrieve the annotations responsible for the false positive counts
221         * 
222         * @return a list of <code>TextAnnotation</code> objects associated with the false positive counts
223         */
224        public List<TextAnnotation> getFPAnnotations() {
225            return fpAnnotations;
226        }
227    
228        /**
229         * Add a false positive <code>TextAnnotation</code>
230         * 
231         * @param fpAnnotation
232         */
233        public void addFPAnnotation(TextAnnotation fpAnnotation) {
234            fpAnnotations.add(fpAnnotation);
235        }
236    
237        /**
238         * Set the false positive <code>TextAnnotation</code> list
239         * 
240         * @param fpAnnotations
241         */
242        public void setFPAnnotations(List<TextAnnotation> fpAnnotations) {
243            this.fpAnnotations = fpAnnotations;
244        }
245    
246        /**
247         * Retrieve the annotations responsible for the true positive counts
248         * 
249         * @return a list of <code>TextAnnotation</code> objects associated with the true positive counts
250         */
251        public List<TextAnnotation> getTPAnnotations() {
252            return tpAnnotations;
253        }
254    
255        /**
256         * Add a true positive <code>TextAnnotation</code>
257         * 
258         * @param tpAnnotation
259         */
260        public void addTPAnnotation(TextAnnotation tpAnnotation) {
261            tpAnnotations.add(tpAnnotation);
262        }
263    
264        /**
265         * Set the true positive <code>TextAnnotation</code> list
266         * 
267         * @param tpAnnotations
268         */
269        public void setTPAnnotations(List<TextAnnotation> tpAnnotations) {
270            this.tpAnnotations = tpAnnotations;
271        }
272    
273    }
```
